# Identification of novel *KMT2D* mutations in two Chinese children with Kabuki syndrome: a case report and systematic literature review

**DOI:** 10.1186/s12881-018-0545-5

**Published:** 2018-02-27

**Authors:** Chengqi Xin, Chun Wang, Yachen Wang, Jingyuan Zhao, Liang Wang, Runjie Li, Jing Liu

**Affiliations:** 1 Stem Cell Clinical Research Center, National Joint Engineering Laboratory, the First Affiliated Hospital of Dalian Medical University, No. 193, Lianhe Road, Xigang District, Dalian, Liaoning Province 116011 China; 2 Department of Neurology, the Second Affiliated Hospital of Dalian Medical University, No.467, Zhongshan Road, Shahekou District, Dalian, Liaoning Province 116027 China; 3 Department of Rehabilitation, Dalian Municipal Women and Children’s Medical Center, No.1,No.3 of Guihuayihao Road, Ganjingzi District, Dalian, Liaoning Province 116000 China

**Keywords:** Kabuki syndrome, *KMT2D*, Novel, Mutation

## Abstract

**Background:**

Kabuki syndrome (KS) is a rare pediatric congenital disorder with multiple congenital anomalies and intellectual disabilities, which is inherited in an autosomal dominant manner. Mutations in *KMT2D* and *KDM6A* have been proven to be the primary cause in most cases of KS.

**Case presentation:**

Here we report two Chinese boys with clinical features of KS referred to our hospital for clinical diagnosis. Next-generation sequencing was performed on MiSeq to analyze the genetic mutations in both patients. In both, two novel de novo mutations in *KMT2D* gene (c.5235delA, p.(A1746Lfs*39) and c.7048G > A, p.(Q2350*)) were detected, both of which were subsequently confirmed by the two-generation pedigree analysis based on Sanger sequencing. A systematic literature review of previously reported mutational spectrum of *KMT2D* was also conducted.

**Conclusions:**

Two novel de novo mutations in *KMT2D* gene were identified and considered to be pathogenic in both of KS patients. Our data adds information to the growing knowledge on the mutational spectrum of KS.

**Electronic supplementary material:**

The online version of this article (10.1186/s12881-018-0545-5) contains supplementary material, which is available to authorized users.

## Background

Kabuki syndrome (KS) (OMIM#147920), also previously known as Kabuki makeup syndrome, or Niikawa-Kuroki syndrome, first reported by Japanese researchers Kuroki [1] and Niikawa [2], is a congenital disorder with multiple congenital anomalies and intellectual disabilities [3]. The cardinal diagnostic manifestations of KS include distinctive facial features, mild-to-moderate intellectual disability, skeletal anomalies, dermatoglyphic abnormalities, and postnatal growth deficiencies [4]. The prevalence of this syndrome is estimated to be 1/86,000–1/32,000 [4].

It has been proved that KS is an autosomal dominant disorder and can be mainly caused by loss-of-function in two different genes-*KMT2D* and *KDM6A*. In 2010, *KMT2D* (NM_003482.3, formerly known as *MLL2*) was identified as the first causative gene in KS patients using whole-exome sequencing [5], which locates on chromosome 12q13. In 2012, *KDM6A* (NM_021140.3) was identified as the second causative gene in three *KMT2D* mutation-negative KS patients [6], which locates on chromosome Xp11.23. These two genes belong to a family of genes called chromatin-modifying enzymes, and act together in the epigenetic control of regulating a diverse set of gene transcriptions involved in embryogenesis and development [7, 8].

Cases of KS patients have been extensively reported from different parts all over the world during past years. However, there are only few cases from China. To the best of our knowledge, only 25 sporadic KS patients have been reported in China [9–18], in which 15 cases performed genetic analysis, including 14 cases with *KMT2D* mutation [15, 17] and 1 case with *KDM6A* mutation [16]. Here, our present study reported two novel de novo mutations of *KMT2D* in two young Chinese boys with KS.

## Case presentation

This study conformed to the Tenets of the Declaration of Helsinki and was approved by the Ethics Board of the First Affiliated Hospital of Dalian Medical University. The CARE guidelines were followed in reporting our cases. Written informed consents from both patients and their parents were obtained before collecting blood samples.

**Fig. 1:**
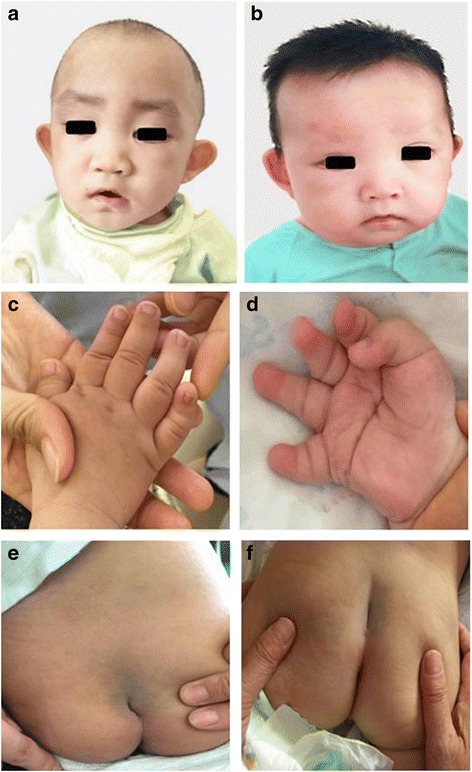
Clinical features of the patients. **a** and **b** showed makeup appearance. **c** and **d** showed short fingers especially for the fifth finger. **e** and **f** showed sacral dimpling

*Patient 1* was the second child (the first one was spontaneous abortion) of healthy, non-consanguineous Chinese parents. There was no family history of genetic disorder. He was born at 36 weeks of gestation due to his mother’s irregular vaginal bleeding. His birth parameters were as follows: weight, 2850 g (25-50th percentile); length, 47 cm (10-25th percentile); and head circumference, 31.5 cm (5-10th percentile). He was admitted to a neonatal care unit for 11 days because of neonatal septicemia and neonatal jaundice. Feeding difficulty and recurrent respiratory tract infection were observed. He showed motor delay and could not turn over until 11 months of age. At 26 months of age, he visited our hospital due to motor and intelligence developmental delay. His height was only 86 cm (3rd-10th percentile). He could not walk alone and his speech was delayed. Facial examination showed long palpebral fissures with lateral eversion of the lower eyelids, arched eyebrows with laterally thinning, depressed nasal tip, lower lip concave, and large ears (Fig. 1a). Cardiac auscultation and ultrasound were normal. He had brachydactyly and prominent finger pads especially for the fifth finger (Fig. 1c). He had sacral dimpling (Fig. 1e). His cognitive disability was mild with an intelligence quotient of 45. In addition, he had abnormal dentitions and abnormal genitourinary system. Urological ultrasound indicated that his right side had ectopic kidney, and left side was narrow and had cyst, and the demarcation between cortex and medulla of both kidneys was not clear. Magnetic resonance imaging of brain and vertebra demonstrated no obvious abnormalities except for the undetectable coccygeal vertebra. Blood routine, urine routine, renal function, thyroid function, electroencephalogram, auditory evoked potential, cardiac ultrasound, electrocardiogram, insulin and levodopa in growth hormone releasing were normal.

*Patient 2* was the second child of healthy, non-consanguineous Chinese parents. There was also no family history of genetic disorder. He was born 40 weeks of gestation by spontaneous vaginal delivery and diagnosed as neonatal hypoglycemia and intrauterine infection. At 6 months of age, he visited our hospital due to motor developmental delay. He could not stand up and sit alone. He had distinctive facial appearance, long palpebral fissures with lateral eversion of the lower eyelids, arched eyebrows with laterally thinning eyebrows, depressed nasal tip, large ears, low hairline, and lower lip concave (Fig. 1b). He also had brachydactyly and prominent finger pads especially for the fifth finger and palm with a straight line across it, and sacral dimpling (Fig. 1d and f). He had joint hypermobility and hypotonia. The examination in blood routine, urine routine and abdominal ultrasound showed no apparent abnormality. Additionally, magnetic resonance imaging of brain suggested cerebellar vermis dysplasia, and urological ultrasound indicated incomplete cryptorchidism on the left side.

As our patients had typical facial features, skeletal anomalies and postnatal growth deficiency, we diagnosed both of them as KS by clinical findings. The basic situation and clinical features were summarized in Table 1. Then we performed molecular genetic testing for the *KMT2D* gene.

**Table 1:** Summary of clinical and genotypic features of 2 Chinese children with KS

Case ID	Patient 1	Patient 2
Gender	M	M
Age at diagnosis (months)	26	5
Elongated palpebral fissures	+	+
Eversion of the lateral third of the lower eyelid	+	+
Laterally sparse eyebrows	+	+
Depressed nasal tip	+	+
Micrognathia	+	+
Abnormal dentition	+	-
Spinal column abnormalities	+	+
Joint hypermobility/dislocation	-	+
Single palmar crease	+	+
Clinodactyly of fifth digits	+	+
Postnatal growth deficiency	+	+
Genitourinary anomalies	+	+
Cerebellar vermis dysplasia	-	+
Hypotonia	+	+
Motor delay	+	+
Feeding problem	+	-
Recurrent infection	+	-
Causing gene	*KMT2D*	*KMT2D*
Exon	22	31
Mutation	c.5235delA	c.7048G>A
Amino-acid change	p.(A1746Lfs*39)	p.(Q2350*)
Mutation type	Frameshift	Nonsense
Novelty	Novel	Novel

Abbreviations: *M*, male; +, present; -, absent

Peripheral blood sample was collected from both patients and genomic DNA was then extracted from blood with a QIAamp DNA Mini Kit (catalog no. 51304, QIAGEN). Library was constructed using TruSight One Sequencing Panel (Illumina, San Diego, CA, USA, which is the largest sequencing panel available and includes 4813 clinically relevant genes), and sequenced on MiSeq platform (Illumina). The sequence was analyzed using ANNOVAR [19], PolyPhen2 [20], Mutation-Taster [21], and various databases, including ClinVar, dbSNP, 1000 genomes, Exome Sequencing Project 6500, and HGMD (Human Gene Mutation Data), were used in our study for screening and annotation of gene variants in accordance with the American college of medical genetics and genomics guidelines [22]. Furthermore, confirmation of the variants found in both patients and analysis of their parental samples were done by Sanger sequencing using standard procedures.

**Fig. 2:**
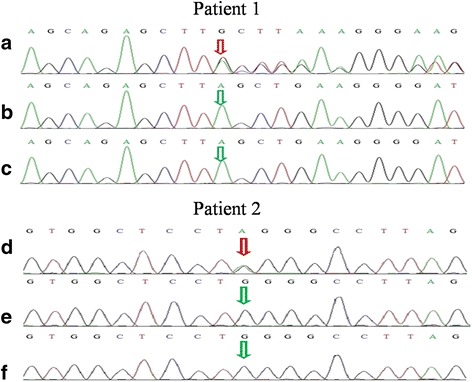
Sanger sequencing results for patients and their parents. **a**-**c** were for patient 1 and his parents, which demonstrated the A deletion (c.5235delA heterozygous mutation, p.(A1746Lfs*39); red arrow) in patient 1, and no deletion at c.5235A (green arrows) in his parents; **d**-**f** were for patient 2 and his parents, which demonstrated the presence of nonsense mutation (c.7948G > A heterozygous mutation, p.(Q2350*); red arrow) in patient 2, and the absence of mutation at c.7948G (green arrows) in his parents

Mutational analysis identified two novel variants in our patients (Table 1). Each patient had one unique individual mutation, and both variants were not detected in their parents. These two de novo mutations included one small deletion leading to a frameshift and premature stop codon (c.5235delA heterozygous mutation, p.(A1746Lfs*39)) from patient 1 (Fig. 2a-c), and one nonsense mutation (c.7048G > A heterozygous mutation, p.(Q2350*)) from patient 2 (Fig. 2d-f).

## Discussion & conclusions

Kabuki syndrome is a rare congenital disease that is characterized by five cardinal manifestations. Its incidence rate is approximately 1 in 32,000 individuals; however, the rate may be underestimated because of misdiagnosis and missed diagnosis. Diagnosis of KS is mainly clinical, based on a combination of distinctive dysmorphic face, intellectual disability, and multiple congenital abnormalities. Our present study reports a new case of two young Chinese boys with KS, both of which were characterized by the most striking facial appearances, skeletal anomalies, visceral anomalies and postnatal growth deficiency. More specifically in detail for both patients, we observed the typical facial appearances, including long palpebral fissures with lateral eversion of the lower eyelids, arched eyebrows with laterally thinning, depressed nasal tip, lower lip concave, and large ears (Fig. 1a-b), and skeletal anomalies, referring to brachydactyly and prominent finger pads especially for the fifth finger and sacral dimpling (Fig. 1c-f), and visceral anomalies, especially for the urogenital system, one with ectopic kidney, and the other with incomplete cryptorchidism. Specially, patient 1 showed feeding difficulty and recurrent respiratory tract infection, and his motor delay was obviously backward. In addition, he had abnormal genitourinary system, urological ultrasound indicating his ectopic kidney, and abnormal dentitions, which are less frequently observed according to previous studies. Patient 2 had cerebellar vermis dysplasia and motor delay, and his urological ultrasound indicated incomplete cryptorchidism on the left side.

**Fig. 3:**
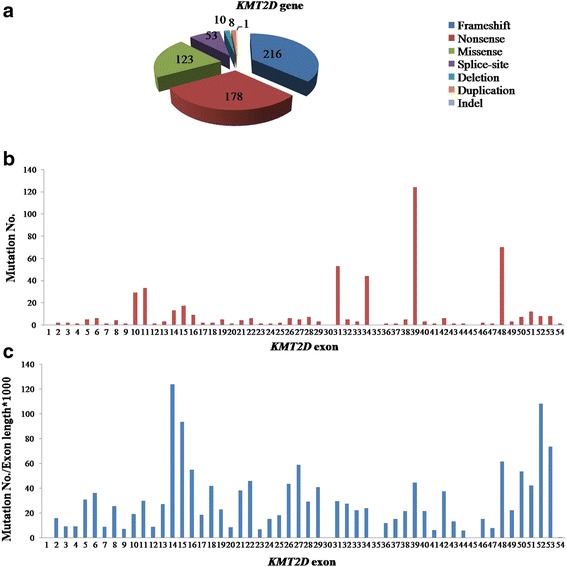
Overview of mutation type and exon distribution of published *KMT2D* mutations. **a**: Mutation types of previously published mutations in *KMT2D*. **b**: Mutation number distribution in *KMT2D* exons. **c**: Mutation distribution in *KMT2D* exons (with exon length taking into account)

Causative genes for KS were identified in 2010 and 2012 for *KMT2D* and *KMD6A*, respectively. Mutations in *KMT2D* were found to be the most common cause and present in 55–80% of KS patients subjected to genetic analysis, and a number of different *KMT2D* mutations have been reported to date. We searched the HGMD database for mutations in *KMT2D*, and performed a detail search for further mutations described in original articles in NCBI PubMed using the key words of “Kabuki syndrome”, “*KMT2D* mutation”, and “*MLL2* mutation” in different combinations, without language restriction. Only articles that were fully available online were used in our analysis (see Additional file 1: Table S1). Then we manually reviewed the obtained articles, including mutation screening studies and molecularly proven case reports, to get the available clinical and molecular information of KS and *KMT2D* mutations. The *KMT2D* mutations were summarized and categorized as “frameshift”, “nonsense”, “missense”, “splice-site”, “deletion”, “duplication” or “indel”. Taking all the reviewed studies together, we found that 589 variants in *KMT2D* gene have been reported so far, including 216 frameshift variants, 178 nonsense mutations, 123 missense mutations, 53 splice-site variants, 10 deletions, 8 duplications and 1 indel (Fig. 3a), in which the majority mutations were frameshift, nonsense and missense, accounting for 36.67%, 30.05% and 20.88%, respectively. We also observed that although the mutations were distributed throughout nearly all exons, there seemed to be some exons in which mutations were mainly located such as exons 39 (23.31%), 48 (13.16%), 31 (9.96%) and 34 (8.27%) (Fig. 3b), which was similar with previous studies [23, 24]. However, we did not see the mutations enrichment phenomenon in the above exons when the length of each exon was taken into consideration (Fig. 3c).

In our present study, one frameshift mutation in exon 22 (c.5235delA heterozygous mutation, p.(A1746Lfs*39)) and one nonsense mutation in exon 31 (c.7048G > A heterozygous mutation, p.(Q2350*)) were identified in *KMT2D* using next-generation sequencing. Considering that all the patients’ parents were not found to carry *KMT2D* mutations, so both the patients harbored two de novo genetic mutations. In summary, we identified two novel de novo *KMT2D* mutations in two Chinese boys with KS, which adds information to the growing knowledge on the mutational spectrum of KS.

## Additional file

Additional file 1: **Table S1.** Summary of published mutations in *KMT2D*. (DOCX 64 kb)
